# Mitochondrial toxins cause widespread downregulation of pathways in X-linked dystonia-parkinsonism patient-derived neurons

**DOI:** 10.1016/j.stemcr.2026.102920

**Published:** 2026-05-07

**Authors:** Karen Grütz, Axel Künstner, Christin Krause, Letizia Santinelli, Sören Franzenburg, Jenny Ghelfi, Anne Grünewald, Raymond L. Rosales, Norbert Brüggemann, Hauke Busch, Christine Klein, Philip Seibler

**Affiliations:** 1 Institute of Neurogenetics, University of Lübeck and University Hospital Schleswig-Holstein, Lübeck, Germany; 2 Medical Systems Biology Group, Lübeck Institute of Experimental Dermatology, University of Lübeck, Lübeck, Germany; 3 University of Lübeck, Lübeck, Germany; 4 Institute of Clinical Molecular Biology, Kiel University, Kiel, Germany; 5 Luxembourg Centre for Systems Biomedicine, University of Luxembourg, Esch-sur-Alzette, Luxembourg; 6 University of Santo Tomas, Faculty of Medicine & Surgery - Neurosciences and Research Center for Health Sciences, Manila, Philippines; 7 Department of Neurology, University Hospital Schleswig-Holstein, Lübeck, Germany

**Keywords:** X-linked dystonia-parkinsonism, iPSC, cortical neurons, transcriptome analysis, DNA damage

## Abstract

The genetic mechanism underlying the neurodegenerative movement disorder X-linked dystonia-parkinsonism (XDP) involves a retrotransposon insertion within the *TAF1* gene. *TAF1* encodes the TATA-box binding protein-associated factor 1, the largest subunit of the basal transcription factor TFIID, which connects transcription activation to the assembly of the RNA polymerase II preinitiation complex at the core promoter of genes. This study investigated how the *TAF1* mutation affects the transcriptomes of XDP patient-derived neurons under basal conditions and in response to mitochondrial toxins. Gene set enrichment analysis revealed that, under basal conditions, patient-derived neurons exhibited predominantly upregulated pathways compared to controls. However, exposure to mitochondrial toxins induced a global shift toward downregulation of pathways in XDP neurons, affecting genome maintenance, epigenetic regulation, adaptive neuronal function, and transcription. Our findings suggest that neurons from XDP patients are more susceptible to mitochondrial stress than controls, leading to widespread transcriptomic downregulation and increased DNA damage.

## Highlights

• Neurons from XDP patients are more vulnerable to mitochondrial stress
• Widespread downregulation of transcriptomic pathways in XDP neurons upon stress
• Mitochondrial stress impacts on DNA repair pathways and DNA damage in XDP neurons

## Teaser

Grütz and colleagues performed comparative transcriptome profiling of neurons derived from patients with XDP at baseline and upon exposure to mitochondrial toxins. Gene set enrichment analysis revealed that pathways were mostly upregulated in XDP patient neurons under basal conditions. In contrast, mitochondrial toxins induced a widespread shift of dysregulated pathways toward decreased expression levels in patient neurons.

## Introduction

X-linked dystonia-parkinsonism (XDP) is a hereditary neurodegenerative movement disorder present in individuals of Filipino ancestry associated with a founder haplotype. Neuropathological analyses of postmortem brains from XDP patients revealed striatal pathology with a progressive loss of medium spiny neurons (Goto et al., 2005) and basal ganglia volume loss (Brüggemann et al., 2016, 2017; Hanssen et al., 2018, 2019, 2023). In addition, changes in white matter microstructures and reduced frontal and temporal cortex thickness have been described (Blood et al., 2018; Brüggemann et al., 2016; Hanssen et al., 2018). The underlying genetic mechanism involves a shared common haplotype identified in all patients, including the disease-causing variant, the SINE-VNTR-Alu (SVA) retrotransposon, within the *TAF1* gene. *TAF1* encodes the TATA-box binding protein-associated factor 1, the largest subunit of the basal transcription factor TFIID, which connects transcription activation to the assembly of the RNA polymerase II preinitiation complex at the core promoter of genes (Bhuiyan and Timmers, 2019).

Notably, genetic modifiers of age at onset in XDP were identified (Laabs et al., 2021). These regions harbor or lie adjacent to *MSH3* and *PMS2*, the genes implicated in modifying age at onset in Huntington’s disease and that likely affect the DNA mismatch repair pathway (Genetic Modifiers of Huntington’s Disease (GeM-HD) Consortium, 2019).

XDP cellular models revealed alternative splicing and partial retention of an intronic sequence proximal to the SVA (*TAF1-32i*) (Aneichyk et al., 2018). This transcript was also present in cells from healthy individuals, albeit in lower amounts than in XDP cells (Pozojevic et al., 2022), but the impact of *TAF1-32i* on the development of XDP remains unclear. Transcriptome analysis from different cell types derived from XDP patients and controls demonstrated the strongest expression changes in iPSC-derived neural stem cells compared to skin fibroblasts and iPSC-derived neurons (Aneichyk et al., 2018). Top terms in each cell type were “response to ER stress” in neural stem cells, “GDP binding” in fibroblasts, and “regulation of cell shape” in neurons.

Multiple common mechanisms have been connected with the onset and progression of neurodegenerative disorders (Bustamante-Barrientos et al., 2023), but if and how these mechanisms affect neurons of XDP patients is still vastly unknown. In Parkinson’s disease (PD), the dysfunction of mitochondria has long been implicated as a core pathogenetic factor (Henrich et al., 2023). Apart from several genetic associations, mitochondrial complex I inhibition was observed to cause a disease phenotype that resembles many features of Parkinsonism in humans and animals (Henrich et al., 2023; Höglinger et al., 2003; Langston et al., 1983). Here, we used mitochondrial complex I inhibitors to perturb neuronal homeostasis and performed comparative transcriptome profiling of mitochondrial stress response in iPSC-derived neurons from XDP patients and healthy controls to identify mechanisms contributing to neuronal loss in XDP.

## Results

### Transcriptome profiling revealed differentially expressed genes in neurons from XDP patients compared to controls

**Figure 1:**
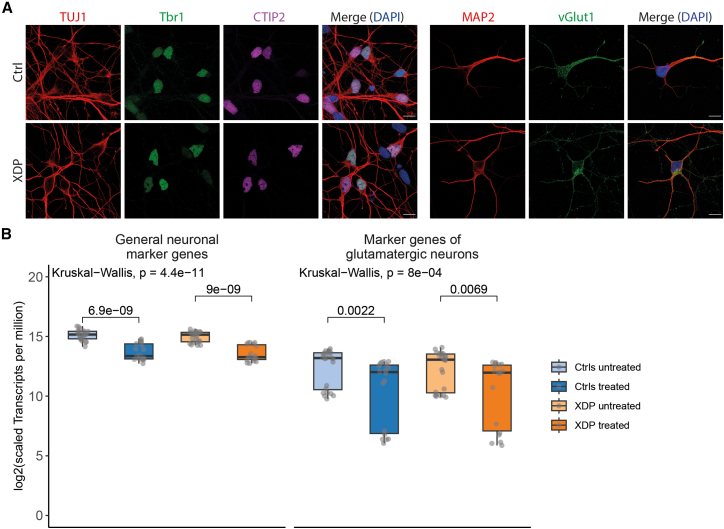
Generation and characterization of cortical neurons derived from iPSC lines of XDP patients and controls (ctrls) (A) Immunofluorescence staining shows the expression of neuronal markers TUJ1 and MAP2, deep-layer cortical markers Tbr1 and CTIP2, the glutamatergic marker vGlut1, and nuclear DAPI. Scales bars indicate 10 μm. (B) RNA sequencing revealed the expression of general neuronal markers (*ENO2*, *MAP2*, and *TUBB3*) and markers of glutamatergic neurons (*DLG4*, *HOMER1*, and *SLC17A7*). The box-scatter plots display the gene expression in transcripts per million of marker sets. Treatment with mitochondrial toxins rotenone and MPP+ caused downregulation of markers. *p* values were determined using Kruskal-Wallis tests, followed by pairwise Mann-Whitney *U* tests for post hoc comparisons. Individual expression levels of marker genes can be found in Figure S1. XDP and control cultures from two neuronal differentiations were analyzed (untreated, *n* = 4 iPSC clones each; treated, *n* = 3 iPSC clones each).

XDP and healthy control iPSCs were differentiated into cortical neurons (Table S1). Immunofluorescence staining confirmed the presence of a population of cells with neuronal morphology displaying the expression of neuronal markers TUJ1 and MAP2, deep-layer cortical markers Tbr1 and CTIP2, as well as the glutamatergic marker vGlut1 (Figure 1A).

To better understand how the XDP mutation impacts neurons under stressed conditions, we performed comparative transcriptome profiling of neurons treated with mitochondrial complex I inhibitors rotenone or 1-methyl-4-phenylpyridinium (MPP+). The treatment conditions were analyzed separately and combined into one group to remove compound-specific effects and increase the statistical power (Table S3). First, we assessed the overall impact of the treatment on neuronal cultures by enrichment analysis of neuronal marker genes in a separate (Figure S1) and combined examination of the toxins (Figure 1B). Upon treatment, we observed significantly reduced expression of general neuronal markers (*ENO2*, *MAP2*, and *TUBB3*) and markers of glutamatergic neurons (*DLG4*, *HOMER1*, and *SLC17A7*) in both groups, neurons from XDP patients and controls, independent of the toxin, suggesting that the treatment had a neurotoxic effect.

**Figure 2:**
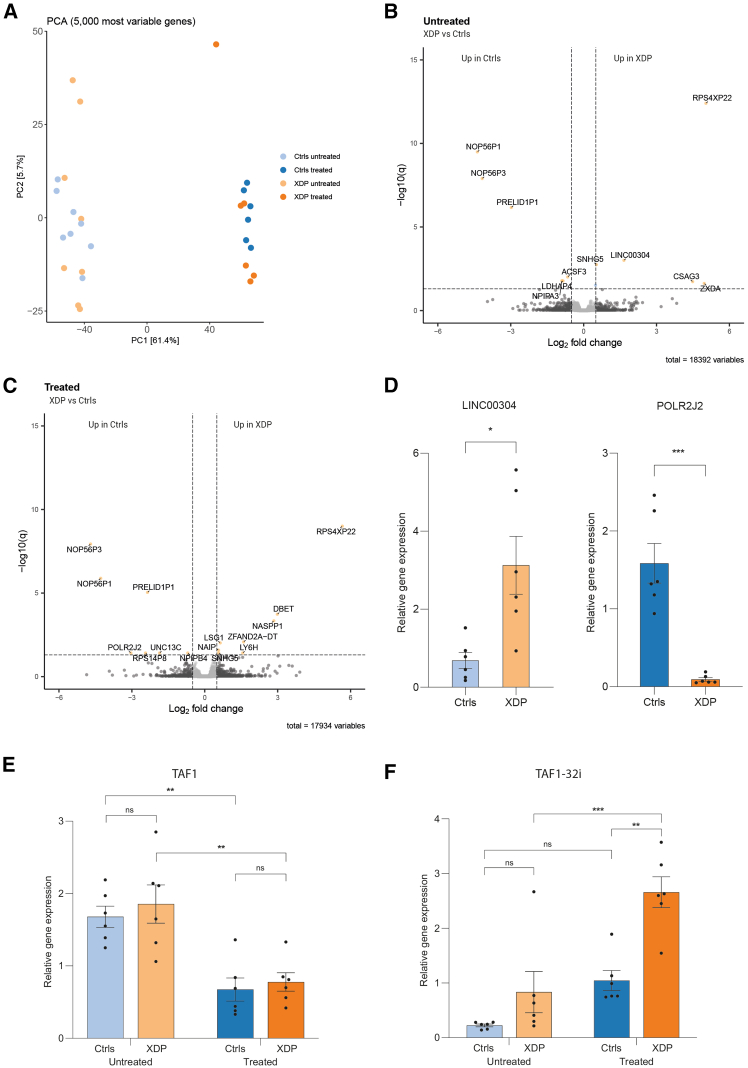
Transcriptome profiling of neurons from XDP patients and controls (ctrls) (A–C) (A) PCA of the top 5,000 most variable genes demonstrates two separate clusters for the untreated and mitochondrial toxin-treated cultures. Volcano plots show differentially expressed genes (*q* < 0.05) between XDP patient neurons and controls for (B) untreated and (C) treated conditions. XDP and control cultures from two neuronal differentiations were analyzed (untreated, *n* = 4 iPSC clones each; treated, *n* = 3 iPSC clones each). (D) RT-PCR expression analysis of long noncoding RNA LINC00304 and *polymerase II subunit J2* (*POLR2J2*) relative to *beta-actin*. Analyzed by unpaired *t* test (^∗^*p* < 0.5, ^∗∗∗^*p* < 0.001). (E and F) RT-PCR expression analysis of canonical *TAF1* and the transcript *TAF1-32i* that contains retention of an intronic sequence proximal to the SVA. Relative gene expression was normalized to *beta-actin* and analyzed by two-way ANOVA and Tukey’s posthoc test (^∗∗^*p* < 0.01, ^∗∗∗^*p* < 0.001; ns, not significant). XDP (*n* = 3 iPSC clones) and control (*n* = 3 iPSC clones) cultures from two neuronal differentiations were analyzed. Error bars display SEM.

Principal component analysis (PCA) on the top most variable genes of our transcriptome data indicates two separate clusters for the untreated and mitochondrial toxin-treated cultures, independent of the disease state (Figure 2A). Importantly, we identified differentially expressed genes (DEGs; q < 0.05) for untreated (11 genes) and treated (15 genes) conditions in the combined analysis, comparing XDP patients and controls (Figures 2B and 2C; Table S3). Validation was performed for two candidate genes linked to RNA polymerase II by RT-PCR (Figure 2D). A significant change in gene expression levels was confirmed for long noncoding RNA (lncRNA) *LINC00304,* upregulated in untreated XDP patient-derived neurons. In contrast, *RNA polymerase II subunit J2* (*POLR2J2*) was downregulated in patient-derived neurons treated with mitochondrial stressors, rotenone or MPP+. Canonical *TAF1* was not found to be differentially expressed when comparing control and patient-derived neurons (Figure 2E). Notably, expression levels decreased in both groups upon treatment. When we quantified the *TAF1-32i* transcript, we observed the opposite effect with treatment, resulting in significantly elevated levels in XDP neurons compared to controls and untreated conditions (Figure 2F).

### Mitochondrial toxins cause widespread downregulation of pathways in neurons from XDP patients compared to controls

**Figure 3:**
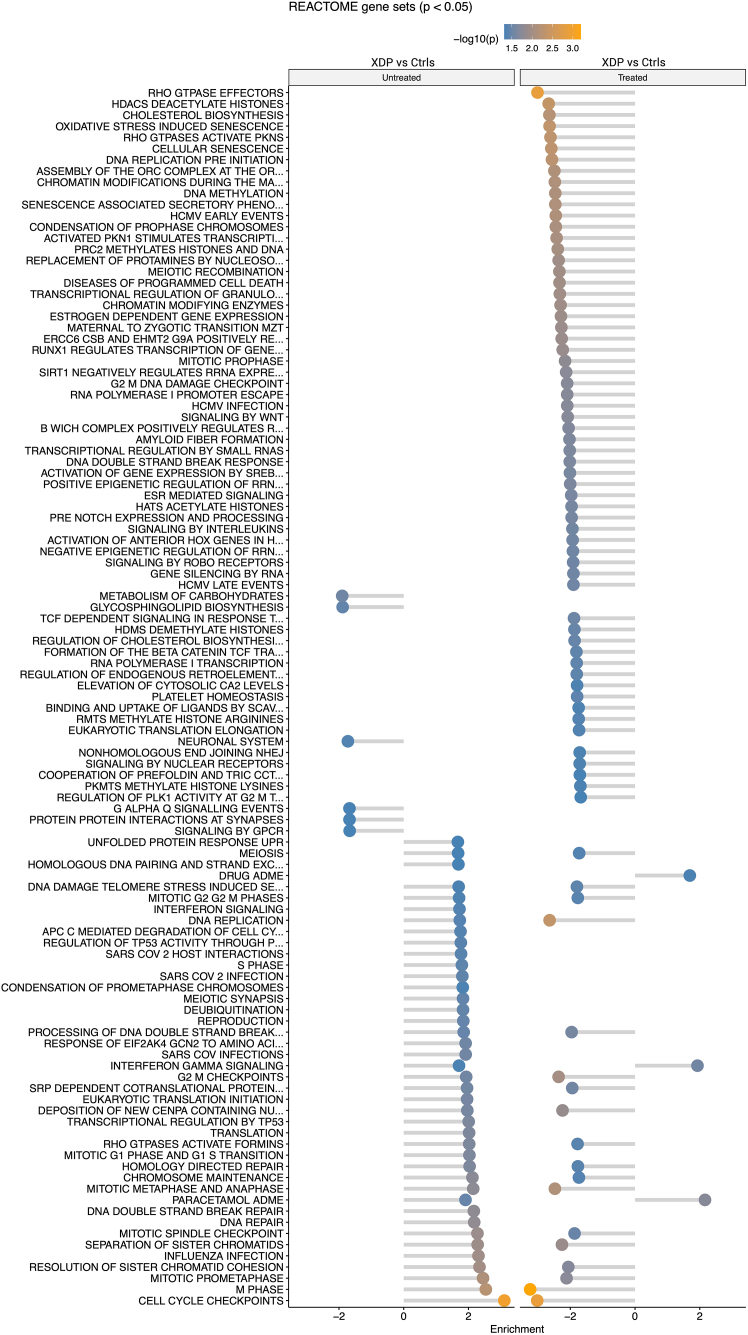
Gene set enrichment analysis Significantly enriched pathways (*p* < 0.05) in neurons derived from XDP patients and healthy controls (ctrls), under both untreated and mitochondrial toxin-treated conditions, are presented in order of increasing *p*-values. XDP and control cultures from two neuronal differentiations were analyzed (untreated, *n* = 4 iPSC clones each; treated, *n* = 3 iPSC clones each).

To obtain cellular mechanistic insight, we performed comparative pathway enrichment analysis of mitochondrial stress response in XDP patient neurons and controls. Under basal conditions, we detected six downregulated and 41 upregulated pathways in XDP patient neurons compared to controls (*p* < 0.05; Figure 3). Pathways of the following biological themes were upregulated in untreated XDP neurons compared to controls: proteostasis and translational control, cell cycle and mitosis, DNA replication and repair, immune response.

Upon treatment with mitochondrial toxins, we observed an overall shift of dysregulated pathways toward decreased expression levels: 79 pathways were downregulated, and only three were upregulated in patient neurons compared to controls (*p* < 0.05). This effect of widespread downregulation was also present in the separate analyses for rotenone and MPP+ (Figure S2). In the combined examination, the downregulated pathways can be grouped in the following biological themes: cell cycle and mitosis, DNA replication and repair, epigenetic regulation and chromatin remodeling, transcription and ribosome biogenesis, senescence and stress response, development and neuronal signaling, RHO GTPase and cytoskeletal control, metabolic and homeostatic pathways.

To further characterize our model on a cellular level, we first assessed mitochondrial function by measuring mitochondrial membrane potential in our neuronal cultures. Rotenone treatment significantly lowered the mitochondrial membrane potential; yet, no differences were observed between control and patient neurons (Figure S3). Next, we explored the mitochondrial DNA (mtDNA) for integrity, deletion load, and copy number using digital PCR. However, there were no differences in the mtDNA analyses between control and XDP neurons (Figure S3).

**Figure 4:**
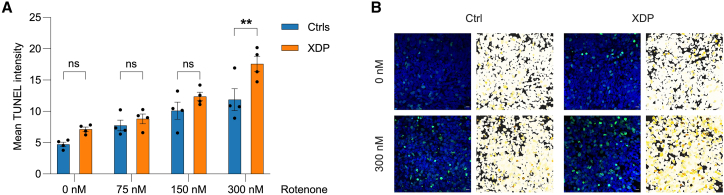
TUNEL assay analysis of neurons from XDP patients and controls (ctrls) (A) The mean TUNEL fluorescence intensities of nuclei were analyzed in cells under basal conditions and upon treatment with different concentrations of rotenone for 48 h. At least three images per cell line and concentration of rotenone were evaluated for analysis (XDP, *n* = 4 iPSC clones; control, *n* = 4 iPSC clones). Error bars indicate SEM. two-way ANOVA followed by Sidak’s multiple comparisons test was performed (^∗∗^*p* < 0.01; ns, not significant). (B) Exemplary immunofluorescence stainings of one control and one patient cell line (untreated and treated with 300 nM rotenone; TUNEL—green; DAPI—blue). Quantification was performed using MATLAB (TUNEL intensities—yellow; DAPI-positive pixels—white). Scales bars indicate 10 μm.

DNA damage repair pathways emerged as one of the themes upregulated in untreated XDP neurons and subsequently downregulated following treatment. We evaluated the proportion of cells exhibiting DNA damage using the TdT-mediated dUTP nick end labeling (TUNEL) assay. We observed an increase in cells with DNA strand breaks in rotenone-treated neurons compared to untreated culturing conditions (Figure 4). Importantly, neurons from XDP patients showed a significantly higher rate of DNA fragmentation upon treatment with rotenone than control neurons.

## Discussion

In this study, we tested iPSC-derived XDP patient neurons for mitochondrial stress-induced cellular pathway disturbances. Transcriptome profiling revealed several DEGs in neurons from XDP patients compared to controls. Our subsequent pathway analysis showed mostly upregulated pathways in XDP patient neurons under basal conditions compared to controls. Treatment with mitochondrial toxins caused an overall shift of dysregulated pathways toward decreased expression levels in XDP patient neurons.

DEGs were validated for two candidates linked to RNA polymerase II. Under basal conditions, lncRNA LINC00304 was significantly upregulated in patient-derived neurons compared to controls. LncRNAs represent the largest group of non-coding RNAs produced from the genome. They are defined as RNA polymerase II transcripts, >200 nucleotides in length, lacking protein-coding potential (Robinson et al., 2020; Zhang et al., 2019). Functional analysis of LINC00304 showed its relation to regulating the cell cycle process, cellular developmental process, and focal adhesion (Zhang et al., 2019). In accordance with that, several cell cycle-related pathways were upregulated in untreated XDP neurons. Upon treatment, most of these pathways were downregulated. *POLR2J2* was identified as one of the top DEGs, significantly downregulated in patient-derived neurons compared to controls. *POLR2J2* encodes a subunit of RNA polymerase II. Its dramatically decreased expression levels could affect the overall transcription efficiency. This finding is interesting in light of the widespread downregulation of numerous pathways we observed in the patients’ neurons after mitochondrial toxin treatment. To rule out RNA degradation as the cause of downregulation, RNA quality was assessed before transcriptomic analysis.

The alterations in RNA polymerase II-related genes and the treatment-dependent transcriptional changes raise the question of whether XDP neurons exhibit a more general transcriptional vulnerability. This hypothesis is supported by the fact that XDP-linked *TAF1* encodes the largest subunit of the basal transcription factor TFIID. While we did not observe a difference in canonical *TAF1* gene expression between control and XDP neurons, we detected a trend toward increased *TAF1-32i* transcript levels under basal conditions and a strong treatment effect resulting in significantly elevated levels in XDP neurons compared to controls and untreated conditions. Altered *TAF1-32i* levels have been suggested previously to contribute to disease manifestation (Aneichyk et al., 2018; Pozojevic et al., 2022).

Furthermore, although we did not observe intrinsic mitochondrial dysfunction, we found that several gene sets involved in DNA damage repair were decreased in mitochondrial toxin-treated patient neurons. Subsequent functional analysis in neurons revealed a significant increase in cells with DNA strand breaks in rotenone-treated XDP patient neurons compared to controls. Vulnerability to mitochondrial stress and accumulation of nuclear and mitochondrial DNA damage are important themes linked to PD (Dölle et al., 2016; Henrich et al., 2023; Sproviero et al., 2025). Rotenone has detrimental effects on mitochondria and can trigger stress that damages lipids, proteins, and DNA (El-Saadi et al., 2022). Interestingly, genetic variants of base excision repair genes and exposure to paraquat and rotenone increase the risk of PD (Sanders et al., 2017). Furthermore, a recent longitudinal analysis of blood samples from PD patients demonstrated a DNA damage signature in patients with more severe progression of motor symptoms (Sproviero et al., 2025). These findings emphasize genotoxic events triggering somatic mutation and cellular dysfunction and suggest their relevance to the pathogenesis of neurodegeneration (El-Saadi et al., 2022). This is in line with the observation that genetic modifiers of age at onset in XDP are associated with genes affecting the DNA mismatch repair pathway (Laabs et al., 2021). Moreover, accurate regulation of RNA polymerase II transcription following genotoxic stress is crucial for the DNA damage-induced stress response (Steurer et al., 2022). This transcriptional regulation might be compromised in XDP due to the *TAF1* SVA insertion. By linking mitochondrial stress sensitivity with impaired DNA damage responses and transcriptional regulation, our data place XDP within a broader mechanistic framework.

Our study also has limitations. While striatal neurons are the main affected subtype, we chose to differentiate into cortical neurons. This method yields a highly homogeneous, well-reproducible neuronal cell population, which is required to reduce heterogeneity in bulk RNA-seq analysis. Further studies are necessary to explore the identified phenotypes also in patient-derived striatal neurons.

Despite these challenges, our findings suggest that neurons from XDP patients are more vulnerable to mitochondrial stress, leading to widespread downregulation of pathways essential for genome maintenance, epigenetic regulation, adaptive neuronal function, and transcription. Our dataset provides a unique resource for further investigations of novel pathways that may be implicated in the underlying pathology of XDP.

## Resource availability

### Lead contact

Further information and requests for resources and reagents should be directed to and will be fulfilled by the lead contact, Philip Seibler (philip.seibler@uni.luebeck.de).

### Materials availability

Materials will be shared with the research community upon reasonable request.

### Data and code availability

RNA-seq data have been deposited at GEO: GSE300875 and are publicly available as of the date of publication. All other data will be shared with the research community upon reasonable request.

## Acknowledgments

This research was funded by the 10.13039/501100001659German Research Foundation (FOR2488) and the Collaborative Center for XDP. A.K. and H.B. acknowledge computational support from the OMICS compute cluster at the University of Lübeck and support by the 10.13039/501100002347Federal Ministry of Education and Research (Germany) (OUTLIVE-CRC; FKZ 01KD2103A).

## Author contributions

Conceptualization, K.G., C.K. and P.S.; methodology, K.G., A.K., L.S., C.K., S.F., and P.S.; formal analysis, K.G., A.K., C.K., S.F., and P.S.; investigation, K.G., A.K., L.S., R.L.R., J.G., A.G., N.B., C.K., and P.S.; writing – original draft preparation, K.G., A.K., and P.S.; writing – review and editing, K.G., A.K., L.S., C.K., S.F., J.G., A.G., R.L.R., N.B., H.B., C.K. and P.S.; funding acquisition, A.K., H.B., C.K., P.S.

## Declaration of interests

C.K. serves as a medical advisor to Centogene and Biogen, received speakers’ honoraria from Bial, and royalties from Oxford University Press and Springer Nature.

## STAR★Methods

### Key resources table

**Table:** 

REAGENT or RESOURCE	SOURCE	IDENTIFIER
**Antibodies**		

Mouse monoclonal anti-TUJ1	Covance	Cat#MMS-435P; RRID:AB_2313773
Rabbit polyclonal anti-Tbr1	Abcam	Cat#ab31940; RRID:AB_2200219
Rat monoclonal anti-CTIP2	Abcam	Cat#ab18465; RRID:AB_2064130
Mouse monoclonal anti-MAP2	Millipore	Cat#MAB3418; RRID:AB_94856
Rabbit polyclonal anti-vGlut1	Synaptic Systems	Cat#135303; RRID:AB_887875

**Chemicals, peptides, and recombinant proteins**		

Matrigel	Corning	Cat#354277
Y-27632	Calbiochem	Cat#688000
Dorsomorphin	Tocris	Cat#3093
SB 431542	Tocris	Cat#1614
Basic fibroblast growth factor	Thermo Fisher Scientific	Cat#13256-029
Brain-derived neurotrophic factor	Peprotech	Cat#450-02
Glial cell-derived neurotrophic factor	Peprotech	Cat#450-10
DAPI Fluoromount-G	Southern Biotech	Cat#0100-20
Rotenone	Sigma-Aldrich	Cat#R8875
MPP+	Sigma-Aldrich	Cat#D048
JC-1	Thermo Fisher Scientific	Cat#T3168
Valinomycin	Sigma-Aldrich	Cat#V0627

**Critical commercial assays**		

CytoTune-iPS Sendai Reprogramming Kit	Thermo Fisher Scientific	Cat#A13780-01
CytoTune-iPS 2.0 Sendai Reprogramming Kit	Thermo Fisher Scientific	Cat#A16517
GenePrint 10 System	Promega	Cat#B9510
TruSeq stranded mRNA Kit	Illumina	Cat#20020594
RNeasy Mini Kit	Qiagen	Cat#74104
Maxima First Strand cDNA Synthesis Kit	Thermo Fisher Scientific	Cat#K1671
Maxima SYBR Green/Fluorescein qPCR Master Mix	Thermo Fisher Scientific	Cat#K0241
*In Situ* Cell Death Detection Kit	Roche	Cat#11684795910

**Deposited data**		

RNA-seq data	This paper	GEO: GSE300875

**Experimental models: Cell lines**		

Human iPSC line: LUEL8360i-5, male, healthy control	deposited to WiCell	LUEL8360i-5
Human iPSC line: LUEL8361i-1, male, healthy control	deposited to WiCell	LUEL8361i-1
Human iPSC line: LUEL8361i-2, male, healthy control	deposited to WiCell	LUEL8361i-2
Human iPSC line: LUEL8356i-2, male, healthy control	University of Lübeck	LUEL8356i-2
Human iPSC line: LUEL5748i-2, male, XDP patient	deposited to WiCell	LUEL5748i-2
Human iPSC line: LUEL5748i-3, male, XDP patient	University of Lübeck	LUEL5748i-3
Human iPSC line: LUEL7756i-2, male, XDP patient	deposited to WiCell	LUEL7756i-2
Human iPSC line: LUEL7756i-4, male, XDP patient	deposited to WiCell	LUEL7756i-4

**Oligonucleotides**		

See Table S2 for primer sequences	N/A	N/A

**Software and algorithms**		

KaryoStudio	Illumina	N/A
FastQC	Andrews, (2010)	https://www.bioinformatics.babraham.ac.uk/projects/fastqc/
salmon	Patro et al., (2017)	https://github.com/COMBINE-lab/Salmon
The R project for statistical computing	R Development Core Team, 2008	https://www.r-project.org/
sleuth	Pimentel et al., (2017)	https://github.com/pachterlab/sleuth
gauge	Luo et al., (2009)	https://github.com/datapplab/gage
GraphPad Prism	GraphPad Software	N/A
MATLAB	MathWorks	N/A

### Experimental model and study participant details

#### iPSC lines and participants

Participants of Filipino origin were recruited to the Department of Neurology at the University of Lübeck and gave signed informed consent to mutation screening and derivation of iPSC lines from skin biopsies. The study was approved by the local ethics committee of the University of Lübeck. Low-passage fibroblast cultures were established from forearm skin biopsies and reprogrammed using CytoTune-iPS and CytoTune-iPS 2.0 Sendai Reprogramming kits (Thermo Fisher Scientific) according to the manufacturer’s protocols (Table S1).

The iPSCs were cultured in mTeSR1 medium (STEMCELL Technologies) on Matrigel (Corning)-coated plates, and mycoplasma testing was regularly performed. Cells were passaged using 0.5 mM EDTA (Sigma Aldrich) in PBS (Thermo Fisher Scientific) every 4–5 days at a 1:10 ratio. The iPSCs were frozen in 90% fetal bovine serum (Thermo Fisher Scientific) and 10% dimethylsulphoxide (Sigma Aldrich) and thawed in mTeSR1 medium supplemented with Y-27632 (10 μM, Calbiochem). Whole-genome single-nucleotide polymorphism (SNP) analysis was performed for iPSC lines and parental fibroblasts on the Infinium OmniExpress-24-Bead Chip (Illumina) and analyzed with the software KaryoStudio v.1.4.3.0 (Illumina), which revealed no karyotypic aberrations (data not shown). All lines were free of bacterial and fungal contamination. Cell line characterization was done between passage numbers 12–15, and differentiation experiments were performed with passage numbers 15–20. Cell line authentication was performed by STR analysis using the GenePrint 10 system (Promega). Clearance of Cytotune Sendai vectors was performed by RT-PCR according to the manufacturer’s instructions (Figure S4). Gene expression levels of pluripotency markers NANOG, GDF3, OCT4, and SOX2 were assessed by RT-PCR (Figure S4; Table S2).

### Method details

#### Neuronal differentiation of iPSC lines

The differentiation into cortical neurons was performed according to a previously published protocol (Shi et al., 2012) with slight modifications (Grütz et al., 2017). iPSCs were plated as single cells. Upon 95% confluency, differentiation was initiated in KSR medium (Knock-Out DMEM/F-12, Thermo Fisher Scientific) supplemented with KnockOut Serum Replacement, L-glutamine (Thermo Fisher Scientific), MEM NEAA (Thermo Fisher Scientific), and 2-mercaptoethanol (Thermo Fisher Scientific) supplemented with dorsomorphin (1 μM, Tocris), SB 431542 (10 μM, Tocris), and Y-27632 (10 μM, Calbiochem). Until day 12 of differentiation, the medium composition was shifted from KSR medium to neural maintenance medium (NMM; 1:1 Neurobasal Medium (Thermo Fisher Scientific) and KnockOut DMEM/F-12 with N2-Supplement (Thermo Fisher Scientific), NeuroCult SM1 Neuronal Supplement (StemCell), L-glutamine, MEM NEAA, 2-mercaptoethanol, and insulin (Sigma-Aldrich)) supplemented with dorsomorphine, SB 431542, and Y-27632. During days 13–17, cells were cultured in NMM, supplemented with basic fibroblast growth factor (20 ng/mL, Thermo Fisher Scientific) and brain-derived neurotrophic factor (BDNF; 20 ng/mL, Peprotech). On day 18, neural rosettes were manually replated and cultured in NMM with BDNF (20 ng/mL), glial cell-derived neurotrophic factor (GDNF; 20 ng/mL, Peprotech), and ascorbic acid (0.2 mM, Sigma Aldrich). On day 23, rosettes were replated again. On day 28, rosettes were dissociated with Accutase and plated in NMM (with BDNF, GDNF, and ascorbic acid). Upon day 43, differentiation factors were withdrawn, and the cells were cultured in NMM for final maturation until day 65–73.

#### Immunofluorescence staining

Neuronal cells were fixed in 4% paraformaldehyde (Sigma Aldrich). Permeabilization and blocking were achieved in PBS containing 4% normal goat serum (Thermo Fisher Scientific), 0.1% bovine serum albumin (BSA) (Sigma-Aldrich), 0.1% Triton X-100 (AppliChem, Darmstadt, Germany), and 0.05% sodium azide (Sigma-Aldrich) for 1 h. Primary antibodies were incubated at 4°C overnight (TUJ1 (Covance), Tbr1 (Abcam), CTIP2 (Abcam), MAP2 (Millipore), vGlut1 (Synaptic Systems)). Secondary antibodies were incubated in PBS with 3% BSA and 0.05% sodium azide for 1 h. Mounting was performed with DAPI Fluoromount-G (Southern Biotech, Birmingham, AL, USA) on glass slides, and images were taken using the LSM 900 (Zeiss, Jena, Germany) confocal microscope.

#### RNA sequencing and data processing

Cells were harvested as untreated and treated, i.e., stressed with rotenone (300 nM) or MPP+ (500 μM) for 48 h. RNA was extracted from whole cell lysates using the RNeasy Mini Kit (Qiagen) according to the manufacturer’s recommendations. RNA quality was assessed by RIN analysis. Only samples with a RIN >6.5 were used for subsequent analyses. The quality of raw RNA sequencing reads was assessed using FastQC (v0.11.5) (Andrews, 2010). Samples were prepared using the TruSeq stranded mRNA kit (Illumina, San Diego) according to the manufacturer’s recommendations with 500 ng input RNA. Resulting libraries were sequenced on HiSeq 4000 (Illumina, San Diego) using 50 bp single-end reads. Transcript-level quantification was performed using salmon (v0.13.0) (Patro et al., 2017), which pseudoaligned reads to the human transcriptome, including both coding and non-coding RNAs, based on Ensembl release 94 (GRCh38); quantification was carried out with 100 bootstrap iterations to enable downstream estimation of technical variance.

#### RT-PCR analysis

According to manufacturers’ instructions, total RNA from cell pellets was isolated using the RNeasy Mini Kit (Qiagen). Reverse transcription of 500 ng of total RNA into cDNA was performed for each sample using the Maxima First Strand cDNA Synthesis Kit (Thermo Fisher Scientific) with dsDNase digest according to the manufacturer’s instructions. Gene expression levels were determined quantitatively on the Lightcycler96 (Roche, Basel, Switzerland) using Maxima SYBR Green/Fluorescein qPCR Master Mix (Thermo Fisher Scientific). The primers are listed in Table S2.

#### Mitochondrial membrane potential and mtDNA analysis

The mitochondrial membrane potential was analyzed using the fluorescent JC-1 probe (Thermo Fisher Scientific). Cortical neurons plated on 12-well dishes (untreated and treated for 48 h with rotenone) were treated with 1 μg/mL of JC-1 for 15 min at 37°C. The cells were washed with PBS, and mitochondrial JC-1 aggregates were measured with a fluorescent plate reader (excitation 528 nm, emission 590 nm). In a sister well, JC-1 fluorescence was measured in the presence of the ionophore valinomycin (1 μM), which destroys the mitochondrial membrane potential, and was subtracted from the data. JC-1 fluorescence was normalized against protein concentration.

The preparation and setup of digital PCR assays to quantify mtDNA integrity, deletion load, and copy number have been performed as published previously (Ghelfi et al., 2025).

#### TUNEL (TdT-mediated dUTP nick end labeling) assay

Cells were stressed with rotenone (75 nM, 150 nM, 300 nM) in NMM for 48 h. Accessible 3′ ends were labeled by FITC-coupled dUTPs. The cells were stained with the primary antibody (TUJ1) for 1.5 h and the secondary antibody for 45 min at room temperature. Cells were incubated with TUNEL solution according to the manufacturer’s protocol (*In Situ* Cell Death Detection Kit, Roche). At least three images were taken for each condition, each consisting of stacks of up to five planes using a confocal laser scanning microscope LSM 710 (Zeiss, Jena, Germany). Each image’s maximum intensity projection (MIP) was subjected to further analysis with ImageJ and MATLAB (Data S1). All TUNEL intensities of DAPI-positive nuclei were averaged and normalized to the area of DAPI-positive nuclei to obtain each sample’s relative TUNEL intensity.

### Quantification and statistical analysis

Statistical analysis for RNA-seq data was performed as follows using R (v4.4.1). Abundance estimates from salmon were imported into the sleuth (Pimentel et al., 2017) R package (v0.30.0) using the wasabi interface (v0.3), and differential expression analysis was performed through pairwise comparisons using likelihood ratio tests to identify significantly regulated genes between conditions. Gene set enrichment analysis (GSEA) was conducted using the gauge (Luo et al., 2009) R package (v2.32.0), utilizing gene sets from the Molecular Signatures Database (msigdf package v2024.1).

The remaining data were analyzed using GraphPad Prism (Version 8, GraphPad Software, La Jolla, USA). T-tests or two-way ANOVAs followed by multiple comparison tests were performed as indicated. The *p* values are illustrated in figures as ^∗^*p* < 0.05, ^∗∗^*p* < 0.01, ^∗∗∗^*p* < 0.001.

## Supplemental information

Document S1. Figures S1–S4, Tables S1 and S2, and Data S1

Table S3. Transcriptome analysis and statistics

Document S2. Article plus supplemental information
