# Supplementary material for: Mitochondrial toxins cause widespread downregulation of pathways in X-linked dystonia-parkinsonism patient-derived neurons
Source: Stem Cell Reports. 2026 May 7;21(6):102920. doi: 10.1016/j.stemcr.2026.102920 (PMC13261877; doi:10.1016/j.stemcr.2026.102920)
Supplement: Document S1. Figures S1–S4, Tables S1 and S2, and Data S1 [file mmc1.pdf]

Stem Cell Reports, Volume *21*

## Supplemental Information

# Mitochondrial toxins cause widespread downregulation of pathways in X-linked dystonia-parkinsonism patient-derived neurons

**Karen Grütz, Axel Künstner, Christin Krause, Letizia Santinelli, Sören Franzenburg, Jenny Ghelfi, Anne Grünewald, Raymond L. Rosales, Norbert Brüggemann, Hauke Busch, Christine Klein, and Philip Seibler**

## Supplemental Material

## Supplemental Figures

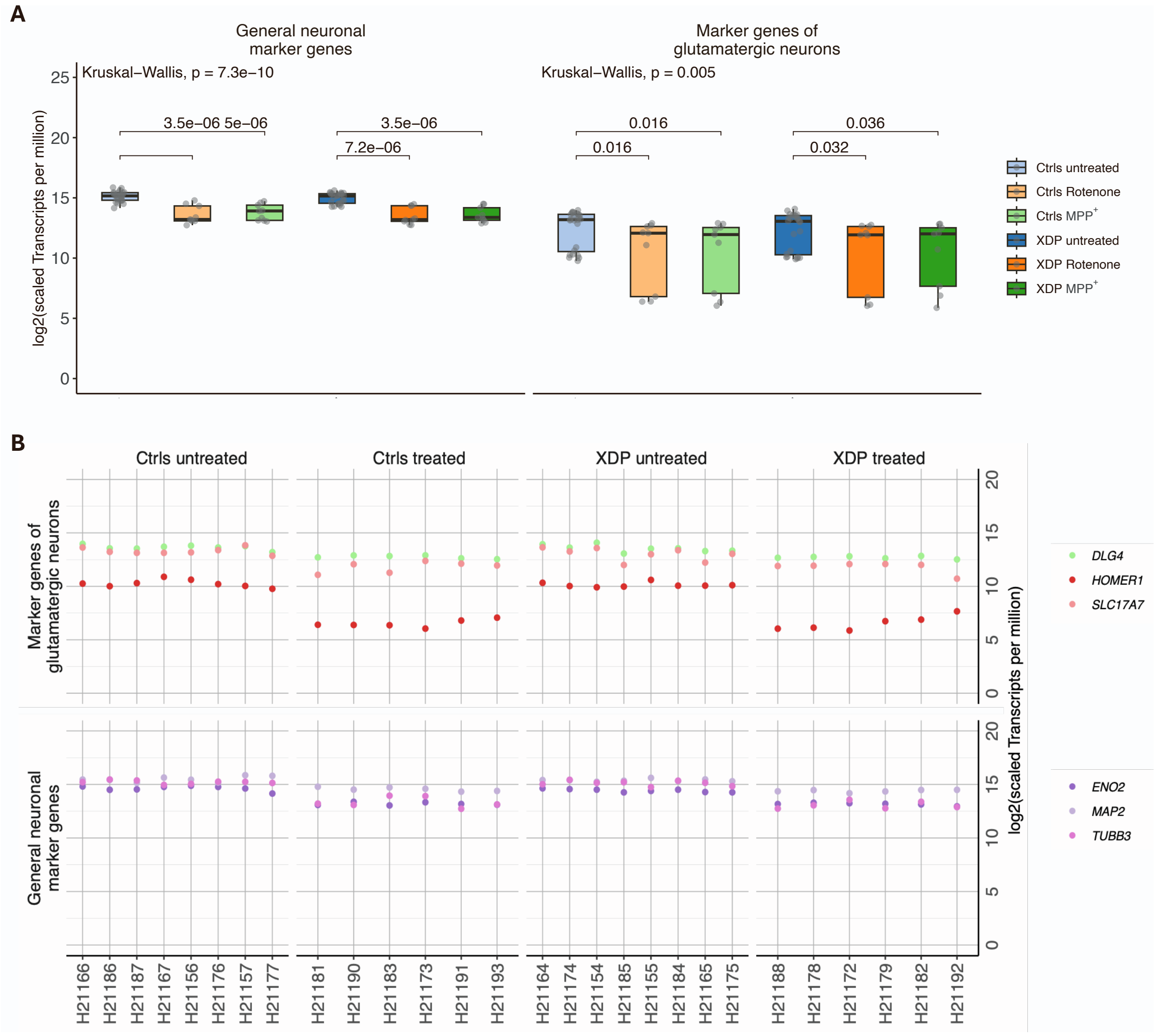


**Figure S1. (A)** RNA sequencing revealed the expression of general neuronal markers (*ENO2*, *MAP2*, and *TUBB3*) and markers of glutamatergic neurons (*DLG4*, *HOMER1*, and *SLC17A7*). The box-scatter plots display the gene expression in transcripts per million of marker sets. Treatment with mitochondrial toxins rotenone (XDP, n = 3 iPSC clones; controls n = 3 iPSC clones) and MPP+ (XDP, n = 3 iPSC clones; controls n = 3 iPSC clones) caused downregulation of markers. P-values were determined using Kruskal-Wallis tests, followed by pairwise Mann-Whitney *U* tests for *posthoc* comparisons. **(B)** Expression levels of general neuronal and glutamatergic marker genes per clone. XDP and control cultures from two neuronal differentiations were analyzed (untreated, n = 4 iPSC clones each; treated, n = 3 iPSC clones each).

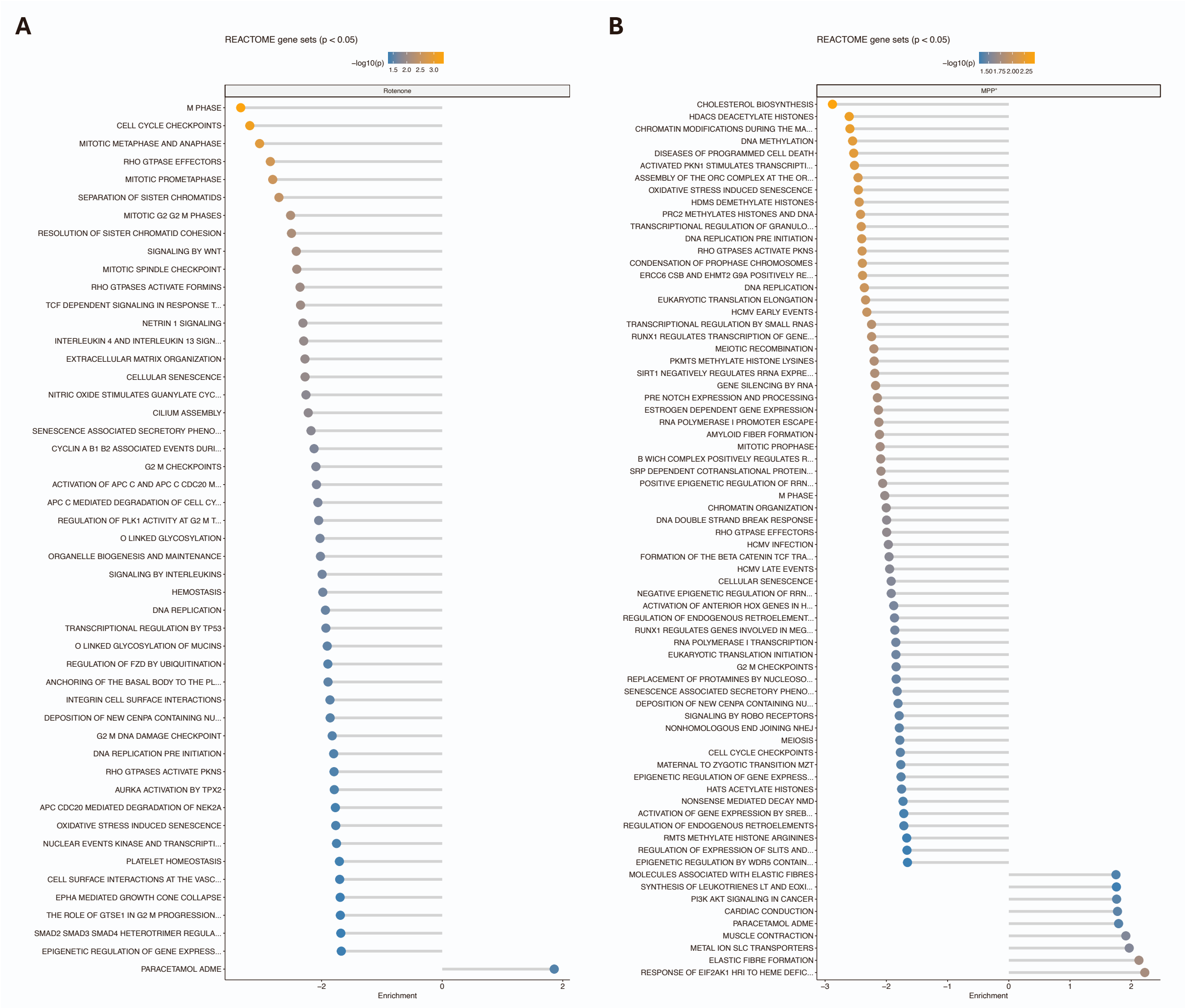


**Figure S2.** Gene set enrichment analysis. Significantly enriched pathways ($p < 0.05$) in neurons derived from XDP patients and healthy controls, under treated conditions. **(A)** Rotenone (XDP, n = 3 iPSC clones; controls n = 3 iPSC clones) and **(B)** MPP+ (XDP, n = 3 iPSC clones; controls n = 3 iPSC clones). Data are presented in order of increasing p-values.

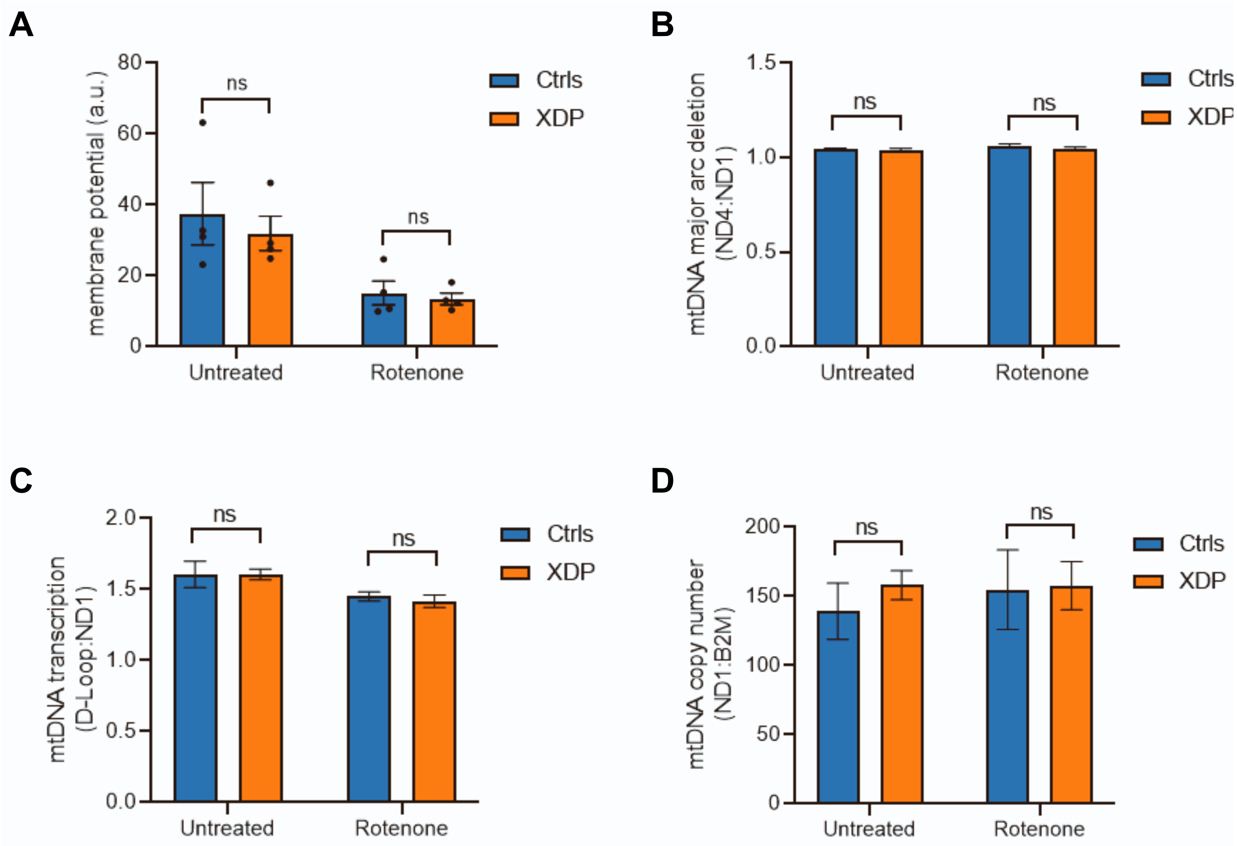


**Figure S3.** Mitochondrial membrane potential and mtDNA analysis. **(A)** Mitochondrial membrane potential was analyzed in untreated and rotenone-treated neuronal cultures (Row factor (treatment) $p = 0.0025$, Column factor = ns). **(B-D)** Digital PCR assays to quantify mitochondrial DNA (mtDNA) deletion load, integrity, and copy number. The ratios of the following gene levels quantified from neuronal cultures allow for interpretation of mtDNA status: **(B)** ND4 / ND1 (indicator of mtDNA deletion load), **(C)** D-Loop / ND1 (indicator of mtDNA integrity and replication) (Row factor (treatment) $p = 0.0111$, Column factor = ns), **(D)** ND1 / B2M (indicator of mtDNA copy number per cell). The cultures were tested untreated and treated with rotenone. XDP (n = 4 iPSC clones), controls (n = 4 iPSC clones). Two-way ANOVA followed by Tukey's multiple comparisons test (ns - not significant). Error bars indicate SEM.

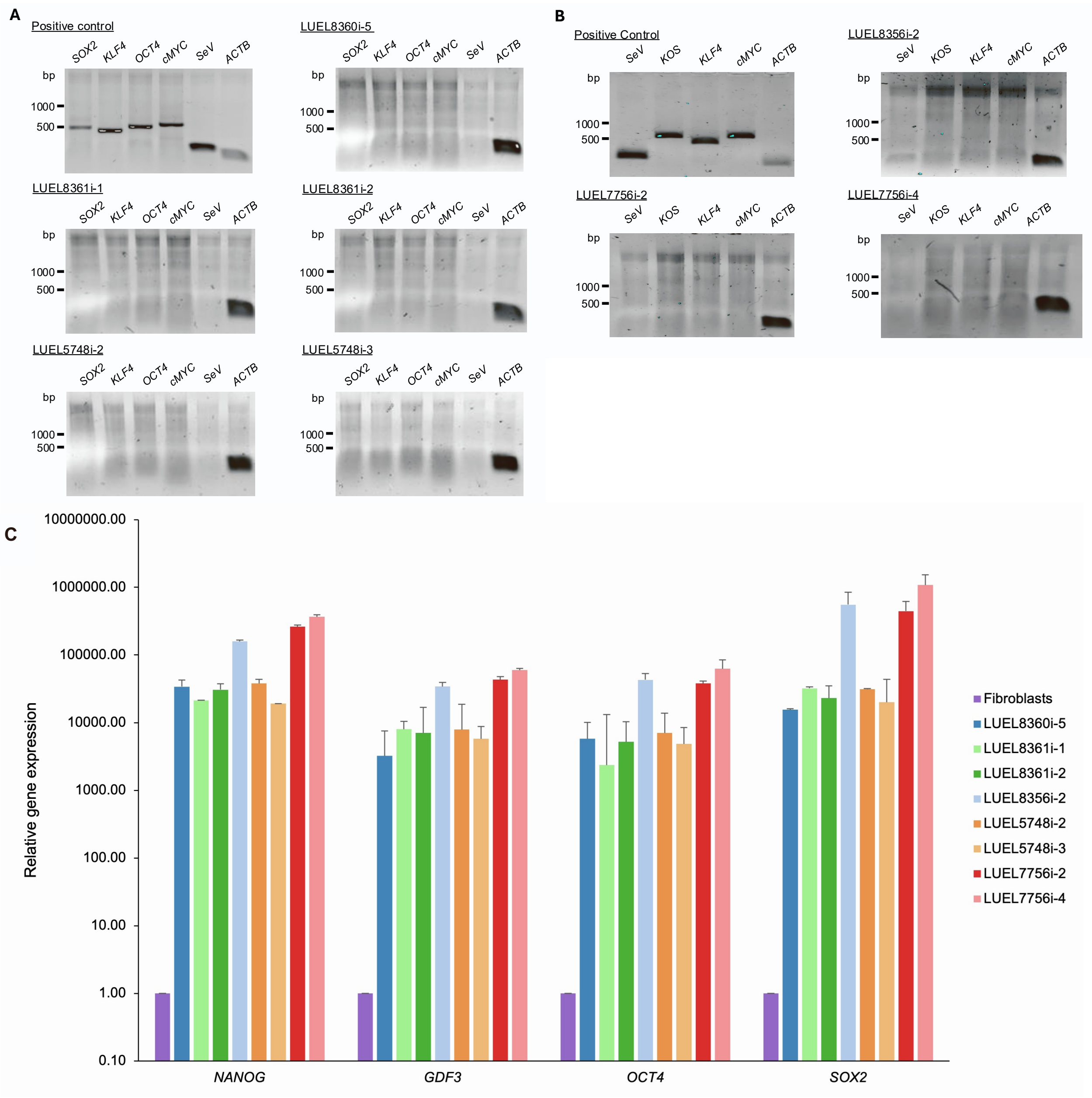


**Figure S4.** Characterization of iPSC lines. **(A+B)** Assessment of Sendai clearance on agarose gels from viral reprogramming factors (OCT4, SOX2, KLF4, cMYC) directly after transduction (positive control) and after ten passaging steps of isolated iPSC clones. **(A)** CytoTune-iPS and **(B)** CytoTune-iPS 2.0 Sendai Reprogramming kits (Thermo Fisher Scientific). Beta-actin (ACTB) served as an internal control. KOS - KLF4, OCT4, SOX2. **(C)** Gene expression levels of pluripotency markers NANOG, GDF3, OCT4, and SOX2 in fibroblasts and iPSC lines relative to ACTB (a loading control) as assessed by quantitative RT-PCR. The values from fibroblasts were set to 1. Error bars indicate SD.

**Supplemental Tables**

**Table S1**: Demographic and phenotypic information of iPSC lines

| iPSC clone ID | Sex | Age at Biopsy | Clinical status XDP | XDP haplotype |
|---|---|---|---|---|
| LUEL8360i-5* | male | 35 | unaffected | wildtype |
| LUEL8361i-1* | male | 34 | unaffected | wildtype |
| LUEL8361i-2* | | | | |
| LUEL8356i-2 | male | 26 | unaffected | wildtype |
| LUEL5748i-2* | male | 42 | affected | hemizygous |
| LUEL5748i-3 | | | | |
| LUEL7756i-2* | male | 35 | affected | hemizygous |
| LUEL7756i-4* | | | | |

* Cell lines have been deposited and are available through WiCell (https://www.wicell.org/)

**Table S2**: Primer sets for RT-PCR reactions

| Gene | Forward 5'-3' | Reverse 5'-3' |
|---|---|---|
| SeV* | GGATCACTAGGTGATATCGAGC | ACCAGACAAGAGTTTAAGAGATATGTATC |
| SOX2* | ATGCACCGCTACGACGTGAGCGC | AATGTATCGAAGGTGCTCAA |
| KLF4* | TTCCTGCATGCCAGAGGAGCCC | AATGTATCGAAGGTGCTCAA |
| OCT4* | CCCGAAAGAGAAAGCGAACCAG | AATGTATCGAAGGTGCTCAA |
| cMYC* | TAACTGACTAGCAGGCTTGTCG | TCCACATACAGTCCTGGATGATGATG |
| KOS* | ATGCACCGCTACGACGTGAGCGC | ACCTTGACAATCCTGATGTGG |
| ACTB[1] | TGAAGTGTGACGTGGACATC | GGAGGAGCAATGATCTTGAT |
| NANOG[1] | TGAACCTCAGCTACAAACAG | TGGTGGTAGGAAGAGTAAAG |
| GDF3[1] | AAATGTTTGTGTTGCGGTCA | TCTGGCACAGGTGTCTTCAG |
| OCT4[1] | CCTCACTTCACTGCACTGTA | CAGGTTTTCTTTCCCTAGCT |
| SOX2[1] | CCCAGCAGACTTCACATGT | CCTCCCATTTCCCTCGTTTT |
| LINC00304[2] | TCTTTTTAAAATGTTGTAGCAATGGA | AGTGGCACATGTCTGTGGTC |
| POLR2J2 | AGGACACCAAGGTACCCAAG | GGTGATGGCGTTGGTAAAGG |
| TAF1[3] | AGAGTCGGGAGAGCTTTCTG | CACAATCTCCTGGGCAGTCT |
| TAF1-32i[4] | GTATAATGATTCAGGAAGTTGCAAG | GTAATGTACCAATATAAATTTCCTGGTTT |

*Primer for Sendai viral genome detection (CytoTune-iPS and CytoTune-iPS 2.0 Sendai Reprogramming kits (Thermo Fisher Scientific))

[1]Park et al., 2008

[2]Zhang et al., 2019

[3]Rakovic et al., 2018

[4]Pozojevic et al., 2022

**Supplemental Datasets**

**Data S1:** TUNEL (TdT-mediated dUTP nick end labeling) intensities were analyzed using the following MATLAB code.

Source code 1: TUNEL_exe.m
Wrapper script to call the getIntensity function.

```
%% --- %% Quantification of TUNEL Intensity
% TODO: Enter path of Dapi binary images (first) and TUNEL grayscale
% images as 8-bit/ PNG
% PATH example: 'C:\Users\UserName\Documents\Dapi\'
% You will get an array 'tunel_Intensity' with values 0 - 255:

[tunel_IntensityNT] = getIntensity('Dapi binary path1', 'TUNEL gray path2');
[tunel_Intensity_2ndConc] = getIntensity('Dapi binary path1', 'TUNEL gray path2');
[tunel_Intensity_3rdConc] = getIntensity('Dapi binary path1', 'TUNEL gray path2');
[tunel_Intensity_4thConc] = getIntensity('Dapi binary path1', 'TUNEL gray path2');
```

Source code 2: getIntensity.m

```
function [ tunelIntensity, cb] = getIntensity( path1, path2 )
%getIntensity calculates the integrated density of a picture pair
%(binary dapi mask and TUNEL, 8-bit)
%   path1 leads to dapi binary masks (0 or 255),
%   path2 leads to TUNEL gray values images, 8-bit

    imgType = '*.png';

    % Load image directory
    imgDapi  = dir([path1 imgType]);
    imgTunel = dir([path2 imgType]);

    % Load images into cell array
    for i=1:length(imgDapi)
        Dapi{i}  = imread([path1 imgDapi(i).name]);
        Tunel{i} = imread([path2 imgTunel(i).name]);
    end

    % Flip columns and lines for better handling
    Dapi  = Dapi.';
    Tunel = Tunel.';

    % For every picture in cell array: Pick Dapi+ pixel (equals 'nucleus')
    % and calculate the mean intensity in TUNEL channel
    for j=1:(length(Dapi))
        nucleus = (Dapi{j,1}(:) == 255);
        tunelIntensity(j,1) = mean(Tunel{j,1}(nucleus));
    end

    % Summation of area
    for j=1:(length(Dapi))
        cb(j,1) = sum(Dapi{j}(:) == 255);
    end
end
```

## Supplemental References


Park IH, Zhao R, West JA, Yabuuchi A, Huo H, Ince TA, Lerou PH, Lensch MW, Daley GQ (2008) Reprogramming of human somatic cells to pluripotency with defined factors. Nature 451:141-146.

Zhang P, Lu Y, Kong Z, Zhang Y, Fu F, Su X, Huang Y, Wan X, Li Y (2019) Androgen-responsive lncRNA LINC00304 promotes cell cycle and proliferation via regulating CCNA1. Prostate 79:994-1006.

Rakovic A, Domingo A, Grütz K, Kulikovskaja L, Capetian P, Cowley SA, Lenz I, Brüggemann N, Rosales R, Jamora D, Rolfs A, Seibler P, Westenberger A, König I, Klein C (2018) Genome editing in induced pluripotent stem cells rescues TAF1 levels in X-linked dystonia-parkinsonism. Mov Disord. 33:1108-1118.

Pozojevic J, Algodon SM, Cruz JN, Trinh J, Brüggemann N, Laß J, Grütz K, Schaake S, Tse R, Yumiceba V, Kruse N, Schulz K, Sreenivasan VKA, Rosales RL, Jamora RDG, Diesta CCE, Matschke J, Glatzel M, Seibler P, Händler K, Rakovic A, Kirchner H, Spielmann M, Kaiser FJ, Klein C, Westenberger A (2022) Transcriptional Alterations in X-Linked Dystonia-Parkinsonism Caused by the SVA Retrotransposon. Int J Mol Sci. 23:2231.
